# Integrating Cognitive Developmental Neuroscience in Society: Lessons Learned From a Multidisciplinary Research Project on Education and Social Safety of Youth

**DOI:** 10.3389/fnint.2021.756640

**Published:** 2021-11-22

**Authors:** Annelinde R. E. Vandenbroucke, Eveline A. Crone, Jan B. F. van Erp, Berna Güroğlu, Hilleke E. Hulshoff Pol, Catherina H. de Kogel, Lydia Krabbendam, Lucres M. C. Jansen, Anne-Marie Brouwer

**Affiliations:** ^1^Developmental and Educational Psychology, Leiden University, Leiden, Netherlands; ^2^Social and Behavioral Sciences, Erasmus University Rotterdam, Rotterdam, Netherlands; ^3^TNO, Soesterberg, Netherlands; ^4^Human Media Interaction, University of Twente, Enschede, Netherlands; ^5^Psychiatry, University Medical Center Utrecht, Utrecht, Netherlands; ^6^Research and Documentation Centre, Ministry of Justice and Security, Den Haag, Netherlands; ^7^Faculty of Law, Maastricht University, Maastricht, Netherlands; ^8^Clinical, Neuro- and Developmental Psychology, Free University, Amsterdam, Netherlands; ^9^Child and Adolescent Psychiatry, Amsterdam University Medical Center, Amsterdam, Netherlands

**Keywords:** cognitive developmental neuroscience, society, integrative method, diversity, team science

## Abstract

Integrating fundamental science in society, with the goal to translate research findings to daily practice, comes with certain challenges. Successfully integrating research projects into society requires (1) good collaboration between scientists and societal stakeholders, (2) collaboration partners with common expectations and goals, and (3) investment in clear communication. Here we describe an integrative research project conducted by a large Dutch consortium that consisted of neuroscientists, psychologists, sociologists, ethicists, teachers, health care professionals and policy makers, focusing on applying cognitive developmental neuroscience for the benefit of youth in education and social safety. We argue that to effectively integrate cognitive developmental neuroscience in society, (1) it is necessary to invest in a well-functioning, diverse and multidisciplinary team involving societal stakeholders and youth themselves from the start of the project. This aids to build a so-called productive interactive network that increases the chances to realize societal impact in the long-term. Additionally, we propose that to integrate knowledge, (2) a different than standard research approach should be taken. When focusing on integration, the ultimate goal of research is not solely to understand the world better, but also to intervene with real-life situations, such as education or (forensic) youth care. To accomplish this goal, we propose an approach in which integration is not only started after the research has been conducted, but taken into account throughout the entire project. This approach helps to create common expectations and goals between different stakeholders. Finally, we argue that (3) dedicating sufficient resources to effective communication, both within the consortium and between scientists and society, greatly benefits the integration of cognitive developmental neuroscience in society.

## Introduction

**FIGURE 1:**
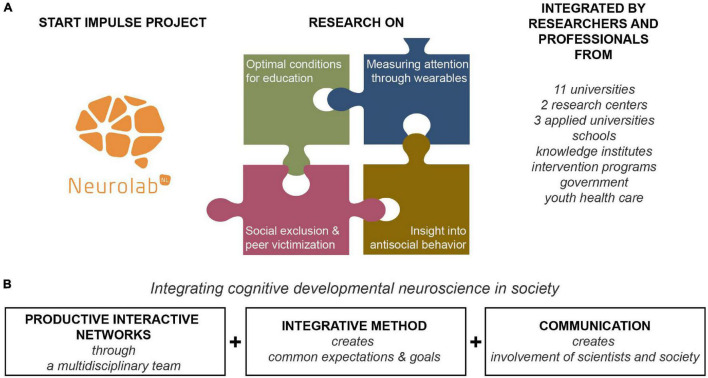
Overview of the NeurolabNL Startimpulse project. **(A)** In four overarching projects, scientists and societal partners worked together to integrate research on optimal conditions of for education (with a focus on motivation and language learning), measuring attention through wearables, insight into antisocial behavior and social exclusion & peer victimization **(B)** The NeurolabNL Startimpulse project integrated cognitive developmental neuroscience in society by forming productive interactive networks, using an integrative method and devoting enough resources to communication.

In the last few decades, our knowledge on neurocognitive development has grown tremendously. Research on brain development, cognitive behavioral development and the combination of these fields has advanced our understanding of the interplay between brain maturation and behavior (Stuss, 1992; Dahl, 2004; Steinberg, 2008; Blakemore and Robbins, 2012; Crone and Dahl, 2012; Crone et al., 2017; Koenis et al., 2018). In this paper, we outline how knowledge from cognitive developmental neuroscience can be integrated in society. Our examples stem from the NeurolabNL Startimpulse research project on adolescents’ academic and social development, conducted by a large Dutch consortium of universities, universities of applied science and societal stakeholders (Figure 1).

To illustrate which types of fundamental knowledge on cognitive developmental neuroscience have been previously implemented in practice, we may consider some of the most well known findings in this field (Box 1). These findings have informed public health policy and education. For example, the accumulated evidence that the brain is not fully matured at age 18 has raised the age of criminal responsibility in several countries (Cornet et al., 2016; SCAAN, 2017; Matthews et al., 2018; Scott et al., 2018; Schmidt et al., 2020). In addition, findings on adolescents’ increased social and emotional sensitivity due to the relatively fast maturing of the limbic area can inform which social-emotional learning activities should be taught in school at what age (Immordino-Yang et al., 2018; Duraiappah et al., 2021), and in general, knowledge about the developing brain can be taken in consideration when designing an optimal learning environment (Trenado et al., 2020). These examples highlight how integrating cognitive developmental neuroscience findings can benefit daily life practices in various domains.

**Box 1. Well-known findings on adolescent brain development.**

Adolescence is marked as the transition period between childhood and adulthood. Its onset is lined up with puberty, which starts around age 9–11 in contemporary society (Dahl, 2004; Crone and Dahl, 2012). Its offset is less well defined. Neurodevelopmental research has shown that cortical brain maturation continues until at least age 21 (Gogtay et al., 2004), white matter maturation reaches it maximum up to age 30 (Yakovlev and Lecours, 1967; Lebel and Deoni, 2018), while cognitive and emotion regulation functions even continue to develop until age 22–25 (Steinberg et al., 2009; Brockmole and Logie, 2013). This is well beyond the age of 18 at which adolescents are commonly agreed upon to enter adulthood in most Western societies. In this paper, we refer to adolescents as youth between the ages of 9 and 21.

One of the most well known findings in cognitive developmental neuroscience is that different brain areas develop at their own speed, and in their own order. Generally, cerebral maturation proceeds from the back to the front of the brain (Stuss, 1992; Gogtay et al., 2004). This means that frontal areas that are involved in the control of behavior are the last to develop. Practically, this suggests that adolescents continue to improve their executive control functions until they are around 21 years of age (Stuss, 1992; Casey et al., 2008; Astle and Scerif, 2009; Crone et al., 2017).

At the same time, the limbic system – a subcortical set of regions that is involved in emotion and reward processing – matures earlier than frontal areas (Casey et al., 2008; Blakemore and Robbins, 2012; Crone and Dahl, 2012), which yields adolescents more susceptible to rewards.

In addition, from puberty onwards, the significance of peer relations increases considerably (Crone and Güroğlu, 2013; Güroğlu, 2021). The combination of peer pressure and the discrepancy in developmental trajectories of the prefrontal versus subcortical brain regions - leading to heightened sensitivity to reward and not yet fully developed behavioral control – leads to dangerous risk-taking and antisocial behavior in some adolescents (Steinberg, 2008; Blakemore and Robbins, 2012). However, for most adolescents, the transition from childhood to adulthood runs smoothly. They take healthy risks that are crucial for development toward independent young adults (such as learning to commute to school with peers instead of parents) and develop mostly prosocial behavior (Dahl, 2004; Crone and Dahl, 2012; Crone and Fuligni, 2020).

### Three Key Challenges

Although the field is advancing, integrating cognitive developmental neuroscience in society remains a challenging endeavor for several reasons outlined below.

#### Productive Interactive Networks

First, setting up good collaborations between researchers and societal stakeholders (i.e., societal partners) is key. If societal partners are not involved in the project, the fundamental research findings often fail to be picked up and integrated in practice (van Atteveldt et al., 2019; Brouwer, 2021). To enhance the chances of creating long-term societal impact, The Royal Netherlands Academy of Arts and Sciences advises to create so-called ‘productive interactive networks’ (KNAW, 2018). This requires time and investment of both scientists and societal partners, who don’t view this as a primary part of their jobs: scientists focus on gaining fundamental knowledge and publications, while societal partners focus on daily practical use. Still, scientists need to invest in gaining knowledge on daily practice and good communication about scientific results, while societal partners need to invest in understanding scientific practice and results. Additionally, even if societal partners are involved in collaborative research projects, they are often only involved in the last phase of the research. At this stage, the research outcomes are usually not aligned to the needs of daily practice, and integration fails. Therefore, to set up successful integrative research, societal partners should be involved in the project from the start. This makes sure that the research project is tailored to the needs, possibilities and limitations of real-life settings (Cornet, 2019; Nauta-Jansen, 2020; Brouwer, 2021).

To tackle this challenge, it’s crucial to have a diverse team of scientists and societal partners. Collaborators with different perspectives and research methods complement each other to create projects that optimally fit with both scientific and societal goals (Cicchetti and Toth, 2006; Ameredes et al., 2015). Today, single disciplinary teams, often with male scientific leaders, still prevail in neuroscience^1^. These teams would benefit from multidisciplinary and diversification. In addition, apart from benefitting fundamental research projects, leadership styles that are more often pursued by women may be particularly suitable for meeting the challenges associated with translating neuroscience to real life, particularly those involving communication and decision-making. A recent report on women leadership showed that female leaders more frequently than male leaders expressed expectations and engaged in participative decision-making (Gipson et al., 2017; McKinsey and Company, 2017). Since integrating cognitive developmental neuroscience in society requires good communication between scientists and societal partners, and setting common goals, we believe that setting up a balanced team of women and men, including more diverse expertise in researchers and societal partners, will benefit integration in practice.

#### Integrative Method

A second challenge is that for successful integration of cognitive developmental neuroscience in society, all parties involved in the project need to have similar expectations. Often, scientists have different goals and expectations than their societal partners (van Atteveldt et al., 2019). To successfully integrate cognitive developmental neuroscience findings, it is therefore important to discuss goals and expectations at the outset of the project.

**FIGURE 2:**
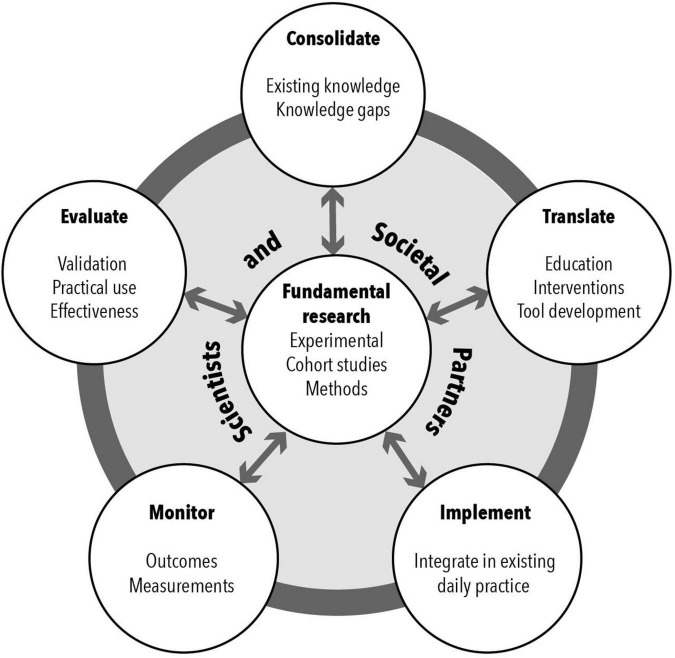
Integrating fundamental research in society at different levels. Fundamental research can have different goals as to how findings may be integrated in society. Depending on the purpose of the project, one can integrate findings by: consolidating knowledge (e.g., exchanging knowledge with stakeholders), translating knowledge (e.g., educational modules), implementing knowledge (e.g., integrate interventions in daily practice), monitoring knowledge (e.g., checking whether scientific knowledge is used properly, and fits with stakeholders’ goals), and evaluating knowledge (e.g., evaluating whether scientific knowledge is effectively used). The process is iterative and dynamic: knowledge is integrated from fundamental research to society and back. Importantly, societal partners and a diverse team of researchers should be involved to guarantee successful implementation. This model is based on Nauta-Jansen (2020).

We argue that integrating cognitive developmental neuroscience in society requires a different than currently standard approach to conducting scientific research. First, when conducting research projects with the goal to integrate the outcomes in practice, societal partners and a diverse research team play a pivotal role. Next, one should think about what the exact goals of the research are and on what level integration can take place (Figure 2): consolidation of (existing) knowledge; translation of knowledge in professional education, interventions or tools; implementation of integrated knowledge; monitoring of the use of this knowledge; or evaluation of the integrated knowledge (Nauta-Jansen, 2020). Importantly, this method of doing research is dynamic, meaning that knowledge is constantly updated and information flows both toward, as well as back from society.

When research projects are designed this way, the ultimate goal is not solely to build knowledge and understand the world better, but also to intervene with real world situations. With this approach, fundamental research does not only directly benefit society, but also scientific theory is enhanced through observation and integration of real-life events. By discussing research with various partners, and learning from studies that are conducted in real-life settings, fundamental theory and laboratory experiments can be greatly improved.

#### Communication

A third challenge relates to communication about research results. For research to be integrated in society, scientific theory and concepts need to be translated such that they are understandable and useful in daily life. This requires skills that scientists are not automatically trained in, since they are used to academic writing.

In addition, it should be clear from the start for both researchers and societal partners what the research may imply on an ethical level: for instance, how will the results change societies’ views on adolescent behavior, youth programs and interventions? What are the benefits and possible pitfalls of using the knowledge in practice (Horstkötter et al., 2017; de Kogel, 2019; Brouwer, 2021)? Involving science communication specialists and ethicists to discuss potential (moral) dilemmas can greatly enhance the communication about complex and sensitive topics, thereby facilitating the use of this knowledge in practice.

### Outline

In the coming sections, we will outline how the NeurolabNL Startimpulse consortium has conducted a collaborative research project in which cognitive developmental neuroscience findings were integrated in society, to improve education and social safety of youth. We will give examples of the studies that were conducted, and highlight on which level integration of knowledge took place (Figure 2). Hereby, we will share our insights into the process and practicalities of integrating cognitive developmental neuroscience in society. When we refer to integration here, we refer to integration of science in society. Integration can of course also take place between different research fields; we see this as one of the success factors for integrating science in society (by setting up productive interactive networks). We will end by making suggestions for best practices to integrate cognitive developmental neuroscience in future research.

## The NeurolabNL Startimpulse Project: Optimal Conditions for Education and Social Safety of Youth

In 2016, the Dutch government invited citizens to ask questions to the scientific community about any topic of their interest. This invitation yielded almost 12.000 questions from the general public, about topics ranging from technology to economy and social sciences. From these questions, 140 were selected and clustered into overarching themes, amongst which neuroscience^2^. Based on this “Dutch Research Agenda,” a funding scheme was designed that supports research projects in which societal partners are directly involved in conducting research and integrating fundamental knowledge in practice^3^. Within this scheme, the NeurolabNL Startimpulse project was designed to gain fundamental knowledge on brain development and its relation to school performance and social behavior, while at the same time creating optimal conditions for integration of this knowledge in society^4^. Below we outline the current outcomes and their integration within the two major sub-themes of education and social safety.

### Education

Education is crucial for adolescents to flourish in contemporary society (Wentzel and Miele, 2016). To create optimal learning conditions, motivation and attention are vital (Wentzel and Miele, 2016). With a team of researchers from universities, research institutes, universities of applied science and teachers in schools, we aimed to further understand academic motivation and attention allocation in groups. Through diverse collaborations, we were able to improve research designs, and develop reflection tools to integrate fundamental knowledge in society.

#### Motivation

Academic motivation is one of the most important predictors of academic achievement and learning (Deci and Ryan, 1985; Fortier et al., 1995; Vallerand et al., 1997). In their influential self-determination model, Deci and Ryan (1985) determined three needs for motivation: autonomy, competence and relatedness. To help teachers reflect on whether they meet these needs, within the NeurolabNL Startimpulse project, we developed a guideline with questions on how teachers integrate these needs using their current teaching methods^5^ (Figure 2: *translation*). Additionally, the guideline provides techniques to improve teaching style with regard to these pillars, together with the possibility to experiment with these styles in the classroom.

To further understand individual differences in motivation, we investigated willingness to invest cognitive effort in schools using a behavioral task (Westbrook et al., 2013). We focused on adolescents, as surveys have shown that motivation for school drops during this phase. In a large sample (*N* = 306), we found that willingness to invest cognitive effort was primarily driven by task-specific ability and to a lesser extent need for cognition (which reflects the degree to which one enjoys thinking). This suggests that adolescents choose strategically how to allocate cognitive resources and do not invest effort when the chance that performance will be successful is small. However, willingness to invest effort was not related to measures of self- and teacher-reported academic motivation. We conclude that in educational practice, increasing the sense of competence and enjoyment may support willingness to invest effort (Kramer et al., 2021).

In the above-described study, we conducted behavioral experiments in schools, and included actual academic achievement measures instead of solely laboratory tasks. Hereby, we were able to link experimental theory to practice. This makes it easier to consolidate these fundamental research findings (*consolidation*). In addition, through this design, we were able to improve our subsequent fMRI studies on the role of brain development in the relationship between cognitive effort discounting and academic motivation (*evaluation*). In addition, in ongoing research we are further investigating dynamic influences on motivation using a diary approach in which adolescents keep track of their motivation for schoolwork (*evaluation*).

#### Attention

A cognitive ability that is closely related to motivation is attention (Wentzel and Miele, 2016). If motivation is low, a student will inevitably lose attention. Vice versa, if attention is distracted, motivation to learn will decrease.

Stimuli that induce cognitive and affective processing elicit specific physiological responses such as a change in heart rate or skin conductance (Libby et al., 1973; Lang et al., 1993; Bradley and Lang, 2000; Brouwer and Hogervorst, 2014). Such responses enable continuous monitoring of attention based on physiology. In particular, electroencephalographic (EEG) responses can be used to identify to which of several sequentially presented stimuli (e.g., beeps or flashes) an individual is focusing her or his attention on, as demonstrated in Brain–Computer Interfaces (Farwell and Donchin, 1988; Nijboer et al., 2008; Schreuder et al., 2010). While monitoring attention in such a way is possible, it requires training of models using context-specific data. In order to obtain responses in real world settings, it also requires assumptions of what forms ‘a stimulus’ in the real world, and knowledge of its exact timing. While these approaches are useful in a lab environment, they are difficult to apply outside the lab in for instance a classroom. Using interpersonal physiological synchrony for monitoring attention bypasses these challenges. Interpersonal physiological synchrony refers to the similarity of signals between individuals over time. A high similarity of EEG signals for individuals who watch and/or hear the same stimuli has been shown to correspond to high outward directed attention (Dmochowski et al., 2012; Ki et al., 2016). For this method, no training of models is required, and it can be used to evaluate attention to natural stimuli. The approach of physiological synchrony allows drawing conclusions on group dynamics, which might potentially be useful in classrooms.

While physiological synchrony in EEG has been demonstrated in the classroom (Dikker et al., 2017), easier, less obtrusive collection and processing of data would make interpersonal physiological synchrony a more suitable measure for attention in the classroom. Therefore, within the NeurolabNL Startimpulse project, we determined whether other physiological measures, namely heart rate and skin conductance, provide similar insights into attentional allocation as EEG (Stuldreher et al., 2020a,b). In this study, participants listened to the same audiobook, mixed with other sounds. Half of the participants were asked to focus their attention on the audiobook; half were asked to focus their attention on the sounds. We showed that indeed, even though EEG is more sensitive, heart rate and skin conductance also contain reliable information on attentional allocation. Classifying a single participant into one of the two instructional groups could be done with 96% accuracy when using synchrony of EEG, and with 73% accuracy when using heart rate and skin conductance. In addition, the reliability of the wearable data that were collected with equipment suitable for real-life settings was similar to the reliability of laboratory equipment (Van Beers et al., 2020), and we observed physiological synchrony in skin conductance and heart rate recorded through wearables in actual classroom settings (Stuldreher et al., 2021). Directly comparing experimental laboratory equipment with equipment suitable for real-life settings form a crucial step in tackling issues around the technical limits to use neurocognitive measures in classroom settings (*evaluation*).

Although scientists often elucidate on possible integration of fundamental findings in practice, it is crucial to engage in a dialog with societal partners themselves to investigate their perspectives on potential benefits and risks of integration. Therefore, in a team of neuroscientists and ethicists, we started qualitative research with focus groups among adolescents to investigate whether using wearable measurements to improve classroom attention and engagement carries their support (*evaluation*). We found that adolescents are in general positive toward using wearables as feedback for teachers, but mainly to be able for them to improve their teaching methods. They are weary of the idea that the devices could be used to monitor individual students. Specifically, students (16/17 years) state that they are old enough to determine whether they pay attention or not: it is their own right to decide. Further research should investigate the sensitivity and added value of using physiological synchrony in realistic cases that potential users – both teachers and adolescents – are interested in.

### Social Safety

One of the most important transitions that adolescents go through is exploration that enables them to gain independence from their parent’s care and yields them to become independently functioning adults (Crone and Güroğlu, 2013; Crone and Fuligni, 2020; see Box 1). While adolescents go through this social-emotional learning process, it is crucial to provide them adequate social safety (Dahl, 2004). By investigating social behavior and brain development with a diverse consortium of researchers from universities, universities of applied science and youth care professionals, we were able to develop educational material and prediction tools to implement knowledge on social safety in practice.

#### Antisocial Behavior

Many adolescents show more antisocial behavior compared to younger children and adults (see Box 1). Antisocial behavior is usually relatively harmless (for example, using a smartphone in class when not allowed), and ceases for most adolescents around age 25 (Liu, 2015; Moffitt, 2018). However, some adolescents remain in life-long trajectories of antisocial behavior that involve behaviors of high severity such as criminal activity. Within the NeurolabNL Startimpulse project, we investigated which neurobiological markers are related to severe antisocial behavior, and how this information can be integrated in practice for diagnostics and treatment to prevent adolescents to become criminally active adults (for an overview of possible implementation in the judicial field, see Cornet et al., 2019).

Neurobiological markers of autonomous nervous system activity, such as heart rate and respiration rate, have been related to antisocial behavior (Zuckerman, 1990; Raine and Liu, 1998; Portnoy et al., 2013; Portnoy and Farrington, 2015; Blankenstein et al., in revision; De Looff et al., submitted). Neuroendocrinological measures such as cortisol and testosterone levels are linked to antisocial and aggressive behavior as well (Alink et al., 2008; Platje et al., 2015; Dekkers, 2018; Blankenstein et al., in revision). However, to study the relation between autonomous nervous system activity and neuroendocrinology with antisocial behavior, often relatively small samples are used. Also, results vary depending on the antisocial behavior that is investigated. Through the extensive collaboration between different universities and youth care facilities, we were able to gain access to and harmonize data from 6 (clinical) samples (Blankenstein et al., 2021). This resulted in a unique dataset of 1,489 participants, displaying none to severe criminal behavior. The findings showed that severe antisocial behavior is linked to neurophysiological measures such as respiration rate, cortisol awakening response and testosterone levels. These findings are of high practical significance as they may be used in addition to psychosocial characteristics to inform diagnostics, risk assessment, psycho-education and eventually (preventive) interventions (Popma and Raine, 2006; Nauta-Jansen, 2020).

Importantly, neurobiological markers cannot be used in their own right, but should be integrated with psychosocial characteristics of individuals. At the moment, an algorithm is being developed to predict antisocial and delinquent behavior from biopsychosocial information within clinical practice (for an example prototype, see^6^; de Ruigh et al., 2021). To implement this algorithm for crime prevention and risk assessment, the current consortium involves partners from Juvenile Justice Institutions and Youth Care to guarantee the feasibility and usability of this tool (*translation*). For example, it is important to consider how therapists choose their intervention programs, such that the knowledge from cognitive developmental neuroscience can add to this. In addition, the assessment of neurobiological markers should be made as easy as possible for both therapists and adolescents, such that they can be used without scientific supervision in daily practice. By conducting the next phases of this project within Juvenile Justice Institutions, and at the same time designing the tools together with different societal partners, implementation of the tools will be more successful.

Even though much is known about the relationship between brain development and antisocial behavior, this information does not automatically reach professionals working with youth. Within the NeurolabNL Startimpulse project, researchers and teachers from universities and applied universities together developed an education package for youth professionals (*translation*). The education package is based on the fundamental knowledge of cognitive developmental neuroscience – specifically during adolescence – that plays a pivotal role in the development of severe and persistent delinquent behavior. In addition, to determine which knowledge is currently lacking in practice, and which knowledge is most important to professionals, teachers and youth themselves, several focus groups were organized among professionals, professionals in training and delinquent youth. Based on this, educational modules on the neurobiology of antisocial behavior, consisting of knowledge clips and accompanying in-depth study assignments for (future) professionals were designed. The modules will become publicly available through websites of the universities of applied science and Dutch governmental bodies, such as the Dutch Youth Institute^7^. This method secures the use of the to-be-developed tools in practice. By collaboration in such a multidisciplinary consortium, the usability and possibility for implementation in practice are greatly enhanced.

#### Social Exclusion and Peer Victimization

Across adolescence, peer relationships become increasingly more important (Crone and Güroğlu, 2013; Güroğlu, 2021). Friendships in this period are among the most important relationships. However, peer relationships do not only involve positive interactions with peers. Unfortunately, less pleasant and adverse interactions with peers, such as social exclusion or victimization, regularly take place in peer contexts. To achieve social-emotional well-being and good mental health, adolescents need to learn how to cope with these adverse peer experiences and related social stress. In addition, to support adolescents in this development, we need to better understand whether some adolescents are more sensitive to negative peer experiences and how this affects their development. More recently, neuroscience research combining examination of peer experiences with neural basis of social interactions has contributed significantly to our understanding of social development (Güroğlu and Veenstra, 2021). Within the NeurolabNL Startimpulse project, we investigated the neural and physiological underpinnings of social exclusion and peer victimization across several lines of research.

In one line of research, we collaborate with two large national prospective population based cohort studies (Generation R: Kooijman et al., 2016; ALSPAC: Boyd et al., 2013; Fraser et al., 2013) to investigate the links between adverse peer experiences and structural brain maturation of children aged 7–10 (on average). These datasets include information on participants’ social networks and peer relationships as well as assessments of DNA methylation and brain structure using MRI. Our findings showed that being bullied is associated with a change in DNA methylation. The gene that shows less methylation has been previously associated with stress in rats (Mulder et al., 2020). This suggests that being bullied affects the adolescents’ stress system. Findings also suggested that white matter microstructure integrity is higher for adolescents who were bullied compared to adolescents who were not bullied. Since white matter integrity increases when the brain develops, we currently interpret these findings to suggest that victimized youth show advanced brain maturation compared to their non-victimized peers (Mulder et al., in prep). Together, these findings add to the growing literature showing that adverse experiences of social stress might have lasting impact on brain development (Teicher et al., 2016; McLaughlin et al., 2019). In the NeurolabNL Startimpulse project, we actively engage societal partners such as teachers and developers of anti-bullying interventions, to pinpoint what these findings might contribute to daily practice (*consolidation*). We do so by organizing regular meetings in which we inform societal partners about our results. Their questions and viewpoints on what these findings imply advance the scientific valorization of our project.

In a second line of research, we investigated the stability of rejection sensitivity, which is a trait that has been associated with peer victimization. Our behavioral study employing longitudinal data on peer victimization and rejection sensitivity has shown that, although victimization and rejection sensitivity are related to each other, they do not enhance each other over time: adolescents who are victimized do not get more sensitive to rejection, and those adolescents that are sensitive to rejection do not get bullied more over time (Kellij et al., in prep). This suggests that certain adolescents are more sensitive to rejection than others, making them more likely to be victimized, but this process does not impact the further development of rejection sensitivity. One crucial open question, however, refers to the impact of chronic experiences of peer victimization over time. To address this question, we have set up a collaboration with an anti-bullying intervention program (KiVa^8^) that is implemented in more than 400 elementary schools nationwide. Schools participating in the program collect data on peer experiences twice across a school year in order to monitor interactions involving bullying and victimization. This unique collaboration between our team of researchers and the anti-bullying program enabled us to access schools with existing longitudinal data on peer victimization, yielding students from these schools a valuable pool of potential participants for our study (*consolidation*). In ongoing research, we are recruiting pupils from these schools for participation in a neuroimaging study that investigates the neural correlates of social cognition in relation to prior victimization experiences.

Our findings, together with previous fundamental knowledge on bullying and social-emotional competence, were used to inform teachers and professionals working with youth through several ways. Together with the Dutch Youth Institute, a publicly available document was constructed to inform teachers and youth professionals about essential scientific knowledge on bullying and adolescents’ social-emotional development^9^ (*consolidation*). In addition, we used this fundamental knowledge to further develop a taxonomy on social cognition (de Mooij et al., 2020). This taxonomy served as a basis to evaluate anti-bullying interventions in schools through a content analysis. For this analysis, programs were scored based on inclusion of markers that should be present based on this literature. Different researchers scored the programs, and compared their scores to reach consensus on the evaluation. Specifically, the evaluation revealed that programs should focus more on the role of social information processing, and the development of emotion regulation strategies and negotiating skills in bullying (van den Bedem et al., in prep). Because of the direct involvement of youth professionals working on anti-bullying intervention programs, the organizations of three widely used intervention programs in the Netherlands cooperated and improved their intervention schemes (*evaluation*). If the intervention program developers had not been involved from the start of the project, this would have been a more difficult trajectory.

## Best Practices

In this paper, we reviewed outcomes of the NeurolabNL Startimpulse project; a national Dutch research program on optimal conditions for education and social safety of youth. The focus of this program was to integrate cognitive developmental neuroscience in society, specifically in education and (forensic) youth care. We identified three main challenges that are often encountered when integrating cognitive developmental neuroscience in society: setting up good collaboration between scientists and societal partners, creating common goals and expectations, and good communication – both within the consortium and between scientists and society. We argue that three factors contribute to overcome these challenges (Figure 1B): (1) forming a well functioning, diverse and multidisciplinary consortium to create productive interactions; (2) using an integrative model to design the research project; and (3) dedicating resources to professional communication. In addition, we highlight the importance of ethics in such as large-scale project, and discuss its translatability to other countries. Below, we outline our lessons learned.

### Forming Productive Interactive Networks

To integrate cognitive developmental neuroscience in society, one of the main challenges is to create productive interactive networks. The necessary basis of such networks is a well-functioning, multidisciplinary team. To tackle this challenge, we have five general recommendations that are elaborated upon below.

#### Early Involvement of Societal Partners

Different stakeholders often have different goals for a project (van Atteveldt et al., 2019). If only one of the stakeholders, often the scientific partner, starts the project, and later on involves other partners, their goals will not be aligned. This will render integration of fundamental knowledge in practice difficult (Cornet, 2019; Brouwer, 2021; Nauta-Jansen, 2020). Throughout the NeurolabNL Startimpulse project, we consulted with different societal partners on the questions they had regarding fundamental research. For example, the fMRI study on chronic peer victimization that is currently being conducted was designed together with input from professionals working with anti-bullying interventions (*consolidation*). For integration of practical tools such as guidelines or algorithms (*translation*), early collaboration is even more important. In our project, for the development of the algorithm that can help predict antisocial and delinquent behavior using neurobiological markers, the involvement of societal partners in juvenile justice settings is crucial for successful integration in practice.

#### Merge Fields – Different Expertise, Ethicists and Diverse Leadership Styles

The success of our integration projects did not only depend on the involvement of societal partners, but also on the diversity of the researchers involved in the project (Cicchetti and Toth, 2006; Ameredes et al., 2015). The topics that were investigated were relatively diverse to start with: motivation, attention, social stress and antisocial behavior. Thereby, the consortium included scientists and applied scientists with various backgrounds, such as in psychology, neuroscience, sociology, criminology and ethics. By regularly meeting and discussing research outcomes, the projects became more integrated with different societal issues, and new collaborations between different fields emerged. For example, wearables were used in a project on social stress at schools, thereby providing a pilot in practice for the wearables on the one hand, and physiological data on social stress on the other hand (*evaluation*).

In addition, we argue that leadership styles that are more often pursued by women, such as expressing expectations and engaging in participative decision-making (Gipson et al., 2017; McKinsey and Company, 2017), greatly contribute to creating a well-functioning team. These leadership styles facilitate setting common goals and expectations between scientists and societal partners, and make sure that the project is tailored to the possibilities and limitations of science and daily practice. Therefore, we believe that setting up a balanced team of women and men, including more diverse expertise in researchers and societal partners, will benefit integration in practice.

#### Involve Youth

Along with creating a diverse research team consisting of adults, youth themselves can greatly enhance successful integration of cognitive developmental neuroscience in society (*consolidation and translation*). Indeed, the knowledge that is integrated is about them, and any intervention that stems from this knowledge should fit with their goals and needs (Gibbs et al., 2018; Grootens-Wiegers et al., 2020; Nauta-Jansen, 2020). Even if the integrated knowledge is perfectly aligned with their environment, if they themselves do not support the intervention that uses this knowledge, the intervention will fail. For example, to reflect on the use of wearables in classes and the use of knowledge on social safety, we conducted several youth focus groups. The input from these focus groups is used to further design follow-up integrative research projects.

#### Knowledge-Brokers

Another successful factor for adequate knowledge communication and integration of fundamental knowledge in practice is to have a person dedicated to this task^10^. Both a researcher’s and a youth professional’s job are not centered on collaborating with each other. A so-called “knowledge-broker” greatly increases the time that can be invested in the collaboration and integration process, thereby enhancing the success of the implementation in practice. For example, in the current project, two knowledge-brokers organized youth panels (*translation, consolidation, and evaluation*), evaluated current interventions based on new scientific knowledge (*evaluation*), and organized networking events (diverse team building). We therefore highly recommend the inclusion of budget for such a position in future grants. Alternatively, if this budget is not available, we recommend having a post-doctoral researcher dedicate some of her time to this task, as it will greatly enhance the collaboration between scientists and societal partners, and between scientists with diverse backgrounds.

#### Networking – Old and New

Finally, the ability – time and moneywise – to network with different stakeholders is crucial for the field to progress. To start with, the research of the teams within the NeurolabNL Startimpulse consortium that had already established connections took off fastest. They had the advantage of already knowing each other’s way of working, and more easily reached out to each other. Therefore, we recommend creating a consortium with at least some relations that are already established. Relatedly, arranging time for the team to get to know each other better, and for the network to grow through match making sessions allows for better integration of different fields and viewpoints. Two of our sub teams tremendously grew and gained more success by active match-making with new partners. By creating a denser and more consolidated network, knowledge can be integrated faster and with more success (*all levels of integration*).

### The Integrative Model

By setting up different types of integration projects (Figure 2), we learned which integrative methods are valued by society and work efficiently. The focus of the NeurolabNL Startimpulse project was to integrate knowledge mainly on the level of consolidation, translation and evaluation. In follow-up projects, we will continue to integrate this knowledge on the level of implementation and monitoring as well. Below, we elaborate on four specific recommendations regarding integrative methods.

#### Educational Material

First of all, many different stakeholders, such as adolescents themselves and youth professionals, mention that transferring knowledge through education is very effective (Cornet, 2019; Nauta-Jansen, 2020; *translation*). This may seem like an obvious path to take, but much of the (to scientists) “basic” knowledge on neurocognitive development has not reached professionals and youth education yet. By developing educational packages that adhere to this basic knowledge – for example on neurobiological markers of antisocial behavior for youth professionals working with criminal youth -, we can reach many people with information that supports professional and personal growth.

#### Research by Practitioners

Throughout the project, we discovered that integration of knowledge also takes place by allowing youth professionals to perform research. In one of our subprojects, a team of teachers in training and scientists conducted studies on motivation for language learning. As part of their teacher training, teachers carried out the experiment at schools, thereby providing the researchers with valuable input on feasibility of the learning method in practice. At the same time, the teachers get acquainted with neuroscience, thereby enhancing their knowledge on brain functioning. By co-creating these projects, researchers can take real-life environmental factors into account, while it helps teachers to develop more efficient teaching environments (Trenado et al., 2020; *integration at all levels*).

#### Evaluate Tools in Practice

To develop tools that can be used in practice, experimental tools almost always need to be adapted (Brouwer, 2021). Often, laboratory experiments are conducted with highly reliable equipment that is not suited for use in daily-life. When developing a tool for practice, stakeholders should be involved from the start to help improve feasibility of the tool. In addition, the tools should be tested to confirm that they are reliable to be used in practice. In the current project, for example, we were able to test wearable devices that measured heart rate and skin conductance in school settings (Thammasan et al., 2020; Stuldreher et al., 2021). This allowed us to test the reliability of these measurements compared to laboratory settings and equipment (*evaluation*). Importantly, without the collaboration within this diverse consortium, this study would not have taken place this easily.

#### Cross-Over From Real-Life Experiments to Laboratory Research

Not only should tools be tested in practice, if knowledge is to be transferred from the laboratory to real-life, the experiments that are performed in the lab should adhere to real-life behavior. By piloting laboratory experiments in real-life settings, one can gain insight into the validity of the constructs that are tested, and whether conclusions from the controlled setting can be extended to daily life (Brouwer, 2021; *evaluation and consolidation*). For example, by conducting behavioral experiments on motivation in school settings using actual academic achievement and motivation diaries, our current fMRI experiments have improved validity.

### Communication

Throughout the project, we focused on clear and effective communication between consortium partners, to interested stakeholders, and to the general public. Three insights aided us in this communication.

#### Include Communication Professionals

In addition to strengthening the interactive productive networks, our knowledge-brokers took on communication within the consortium and the dissemination of research results. To do so, they organized several activities for the consortium to get together, to disseminate knowledge to a broad audience (*consolidation*) and served as editor for educational material (*translation*). Importantly, these knowledge-brokers were skilled at translating scientific findings to a broad audience, because of their own scientific background and direct collaborations with societal partners.

In addition, by including ethicists on different research projects, moral dilemmas and potential pitfalls of communication were discussed. For example, the implications of education on neurobiological markers of antisocial behavior, and the use of wearables to track attention were discussed (Horstkötter et al., 2017; de Kogel, 2019; Brouwer, 2021). This enhances the success of translating these results to society (*translation*).

#### Use Existing Platforms

Translating knowledge by updating or adding information to already existing communication platforms is more effective than setting up new communication pathways. For example, at the outset of the NeurolabNL Startimpulse project, we planned to design a new website for youth professionals to share information about brain development and bullying. Instead, throughout the project, we found that the Dutch Youth Institute already had quite extensive communication channels and were interested to update their information regarding bullying and social emotional capacities. By integrating our knowledge, we were able to communicate to youth professionals in a faster and more efficient manner (*consolidation and translation*). This example shows the benefits of a well-functioning interactive productive network, illustrating that long-term societal impact might be realized through parties that were not part of the initial research consortium.

#### Target Specific Stakeholders

Although time-consuming, one of the most effective ways to consolidate knowledge is to organize workshops or lectures for stakeholders that directly benefit from the scientific knowledge. In the NeurolabNL Startimpulse project, for example, researchers gave lectures at schools and organized workshops for educational modules for teachers. According to our collaborating teachers and education coaches, this way, knowledge best reaches the interested stakeholders, and makes them more likely to use the information in practice. However, since scientists in general don’t have much time to spend on giving lectures for societal partners, this suggestion feeds back into the recommendation of including communication professionals in integrative research projects.

### Ethics and Data Privacy

Through our extensive collaboration with different partners, datasets were shared between scientists, as well as between societal partners and scientists. This made data privacy a very important topic in many of our studies. To make sure that privacy regulations were upheld, we consulted with ethical, juridical and data science experts, who advised on reusing data. For example, before sharing data, agreements regarding who had access to data and where it was stored were signed. In addition, as is common in experimental developmental cognitive neuroscience research, we only used data for which participants signed informed consent, stating that their data was made anonymous and only used for research purposes.

### Translatability to Other Countries

Since the current project was conducted in the Netherlands, it had a favorable start with respect to the possibility to integrate science in society: in the Netherlands, there is already a well-established connection between science and society, and a government that supports the integration of science in society. This may have led to an emphasis in the current project on setting up good collaborations and the integrative method. We acknowledge that in other countries, this might be different, and starting integration at the level that we describe here is not possible. If the logistics and culture are not supportive of science, or the integration of science in society, communication between scientists and society is even more important, and forms the starting point for future collaborations. Still, if there is a wish to integrate science in society, we argue that the three basic needs for integration – early collaboration, choosing integrative methods, and communication – are similar for all countries.

## Conclusion

In the last decades, our knowledge on cognitive developmental neuroscience has advanced such that it becomes increasingly more possible to integrate this knowledge in society. However, successfully integrating research findings remains a challenge: fundamental theories and experimental methods cannot be translated to practice one-on-one. Based on the research within the NeurolabNL Startimpulse project, we learned that (1) good collaboration between scientists and societal stakeholders, (2) common expectations and goals between partners, and (3) investment in clear communication are key in successfully integrating cognitive developmental neuroscience in society (Figure 1B). We propose to (1) conduct research projects in consortia in which researchers from different backgrounds and societal partners work together in diverse teams, creating interactive productive networks; to do so, it is important to involve societal partners at the start of the project, such that goals and expectations of scientists and societal partners are aligned. Next, the team should consist of researchers and societal partners from different institutional types with different expertise and research methods. Relatedly, we advocate having a diverse team to promote the integration of different leadership styles and communication methods. Related to the topic of this special issue ‘Women in Neuroscience,’ we believe that leadership styles that are more often pursued by women, such as expressing expectations and participative decision-making, contribute to optimal transfer of knowledge, both within the consortium, and between science and society. Next, we propose to (2) use an integrative approach in conducting research (Figure 2). To effectively do so, the research goals and level of integration should be clearly defined at the start of the project. Last, we propose to (3) dedicate sufficient resources to communication, both within the consortium, and to interested stakeholders and the general public. By adhering to these three recommendations, we believe that fundamental knowledge can most effectively be integrated into practice, while at the same time, scientific theory is strengthened by knowledge embedded within society.

## Author Contributions

All authors wrote the manuscript together. A-MB initiated the submission. AV coordinated the process and was the main writer. All authors contributed to the article and approved the submitted version.

## Conflict of Interest

The authors declare that the research was conducted in the absence of any commercial or financial relationships that could be construed as a potential conflict of interest.

## Publisher’s Note

All claims expressed in this article are solely those of the authors and do not necessarily represent those of their affiliated organizations, or those of the publisher, the editors and the reviewers. Any product that may be evaluated in this article, or claim that may be made by its manufacturer, is not guaranteed or endorsed by the publisher.

## Footnotes

1
https://www.frontiersin.org/research-topics/19592/women-in-neuroscience#overview

2
www.neurolab.nl/en

3
https://www.nwo.nl/en/researchprogrammes/dutch-research-agenda-nwa

4
www.neurolab.nl/en/startimpulse

5
https://lic.avans.nl/service.lic/publicaties/motivatie-motor

6
https://architecta.shinyapps.io/PredictingYouthReoffending/

7
www.nji.nl/english

8
https://www.kivaschool.nl/

9
www.nji.nl/nl/Kennis/Dossier/Pesten

10
https://www.nro.nl/nieuws/onderzoek-en-onderwijspraktijk-verbinden-vier-randvoorwaarden-voor-een-succesvolle?utm_campaign=nieuwsbriefjan20&utm_medium=nieuwsbrief&utm_source=nieuwsbriefjan20
